# Assessment of Cardiovascular Risk Factors Among University Students: The Gender Factor

**DOI:** 10.4021/cr198e

**Published:** 2012-07-20

**Authors:** Mohammad Y. Gharaibeh, Karem H Alzoubi, Omar F. Khabour, Lubna Tinawi, Rawan Hamad, Esraa F. Keewan, Sulaiman K. Matarneh, Mahmoud A. Alomari

**Affiliations:** a Department of Medical Laboratory Sciences, Faculty of Applied Medical Sciences, Jordan University of Science and Technology, Irbid, Jordan; b Department of Clinical Pharmacy, Faculty of Pharmacy, Jordan University of Science and Technology, Irbid, Jordan; c Department of Physiology, Faculty of Medicine, Jordan University of Science and Technology, Irbid, Jordan; d Department of Allied Medical Sciences, Faculty of Applied Medical Sciences, Jordan University of Science and Technology, Irbid, Jordan

**Keywords:** CV disease, Risk factors, University, Students, Jordan

## Abstract

**Background:**

Evidence indicates that the pathophysiological process of cardiovascular (CV) disease begins at early age, though the manifestations of the disease do not appear until middle age adulthood. Risk factors for CV disease, particularly lipoprotein profiles, are affected by physiological abnormalities, and lifestyle related issues. To evaluate prevalence of CV diseases risk factors among university students and to investigate relation between number of risk factors and body anthropometric, hematological and biochemical indices parameters.

**Methods:**

In this cross sectional study, 348 students were randomly recruited. Blood glucose, cholesterol profile (total, HDL, and LDL cholesterol), and triglyceride were measured using standard protocols. Physical activity (PA) level was assessed using the short-form Arabic version of the International Physical Activity Questionnaires (IPAQ).

**Results:**

The most commonly encountered CV disease risk factor was low levels of HDL-C, followed by physical inactivity, high levels TG, and obese BMI. When stratified by gender, females were less likely to have low HDL-C, and high TG, whereas, males were more likely to have overweight or obese BMI (P < 0.001). About 49% of the participants had at least one CV disease risk factor, where as the prevalence of having one, two and three or more CV disease risk factors were 35.7%, 9.3% and 4%, respectively. Additionally, the number of CV disease risk factors showed strong positive correlation with increases in body fat and bone percentages, glucose, total cholesterol, TG, LDL-C, BMI, and WHR (range of R^2^: 0.17 to 0.603). On the other hand, physical activity, percentages of body water and muscle, HDL-C showed inverse strong correlation with cardiovascular risk factors (range of R^2^: -0.239 to -0.412).

**Conclusions:**

Results indicate the high prevalence of CV disease risk factors among university students, and stress the need for early intervention programs to counteract these risks.

## Introduction

Cardiovascular (CV) diseases are the leading cause of mortality and morbidity in the world even in the developing countries [1]. The manifestations of the diseases increase in middle-aged adults, however, many studies reported signs of atherosclerotic pathogenesis in younger adults and children as young as 5 - 6 years old [2-4].

Serum cholesterol ≤ 200 mg/dL, no history of diabetes, not diagnosed previously with myocardial infarction and no baseline ECG abnormality have been recognized as favorable CV risk profile [5]. Meanwhile, elevated serum cholesterol, diabetes, and obesity have been identified as major risk factors [5]. The relationships of these risk factors with coronary, cerebral, and peripheral vascular diseases have been described as strong, continuous, graded, consistent, independent, predictive, and etiologically significant [6].

Regular physical activity, on the other hand, increases exercise capacity and plays a role in primary and secondary prevention of CV diseases [7-8]. In addition, physical activity is also considered the most important modifiable risk factor of CV diseases [9]. For example, physical activities promote controlling blood lipid abnormalities, diabetes, and obesity. On the other hand, sedentary lifestyle is associated with high risk of coronary, cerebral, and peripheral vascular diseases and all-cause morbidity and mortality among both genders at all ages [10]. It is estimated that more than 2 million deaths annually in the world are attributed to physical inactivity [11]. Therefore, implementing physical activity programs in schools, colleges, community centers, and even at job sites is recommended [12].

The prevalence of each risk factor for CV diseases might differ among different countries and populations, which will affect the implementation of intervention programs. For example, a study that examined physical activity in twenty countries showed wide ranges in the frequency of physical inactivity with 7-43% and 6-49% prevalence among men and women respectively [13]. Similarly, a study that examined CV diseases risk factors in several countries from the Middle East and North Africa showed wide variations in the prevalence of obesity, diabetes, hypertension, hyperlipidemia, smoking and physical inactivity among examined populations [14]. For successful prevention programs, several studies have suggested the importance of targeting young adults and identification of risk factors in this population [15-20]. Risk factors of CV diseases in young adults from various countries including Columbia [16], Chile [19], Brazil [18], Japan [17], Serbia [21], and Portugal [15] has been reported, however, not yet from Arab countries. In this study, we evaluated the prevalence of CV disease risk factors, and the association of these factors with body anthropometric, hematological and biochemical indices among male and female university students in Jordan.

## Materials and Methods

### Subjects

This cross sectional study was conducted at the Jordan University of Science and Technology (JUST) campus during the period from June until September, 2009. Participants were recruited through student's wall advertisements that were posted all over the university campus. Those who responded were invited to a special laboratory to complete a study form that asked about simple demographic information such as age and gender. Thereafter, anthropometric measurements were carried out for the participants. Students were then asked to fast overnight for blood collection. All subjects were healthy individuals 18 to 23 years old. Students with chronic diseases or those taking medications were excluded from the study. After a full explanation of the study details, each participant signed an informed consent approved by the IRB of JUST in accordance with the principles described in the Declaration of Helsinki, including all amendments and revisions. Data were aggregated and stored in a secure place with access only to the authors and the investigators, to ensure confidentiality.

### Cardiovascular risk factors

Blood glucose, cholesterol profile (total, HDL, and LDL cholesterol), and triglyceride were measured in each participant as described previously [22]. In brief, blood samples were collected in the morning after overnight fasting from the cubital vein in plain and EDTA tubes, and serum/plasma were stored at -80 °C until used. HbA1C was measured from whole blood via immunological assay of hemoglobin A1C using automated clinical chemistry analyzers (Roche, Basel, Switzerland). Glucose, total cholesterol, LDL-C, HDL-C, TG) were measured photometrically using enzymatic assay Kits. CBC and other blood parameters were measured using Automated Hematology Analyzer, Sysmex Xt 2000i (Sysmex Corporation, Kobe, Japan). The result was expressed as mmol/L.

### Anthropometric measures

Body weight and height were measured to calculate body mass index (BMI) as weight in kg/height in meters^2^. After measuring the waist and the hip, as the greatest circumference around the pelvic, the ratio (WHR) was calculated. Standard classification of BMI and waist circumference cut off values was adopted as previously described [23].

### Total Physical activity

Physical activity (PA) level was assessed using the short-form Arabic version of the International Physical Activity Questionnaires (IPAQ). All individuals in the study were given the questionnaires after detailed description of the questions. The IPAQ is 7 questions designed to evaluate participation in walking, moderate, and vigorous PAs and expressed in MET·min·wk^-1^. Subsequently, total PA was calculated as walking + moderate + vigorous and categorized into low (> 600 MET·min·wk^-1^), moderate (600 - 1500 MET·min·wk^-1^), and high (1500 MET·min·wk^-1^) and assigned the values 1, 2, and 3, respectively [24] (http://www.ipaq.ki.se/). The IPAQ was recently validated in several international studies [25-27].

### Calculation of Risk factors

The following were considered as risk factors: blood glucose of > 5.89 mmol/L, Total cholesterol of ≥ 5.2 mmol/L, Triglyceride of > 1.7 mmol/L, HDl of ≤ 1 mmol/L, LDL of ≥ 3.4 mmol/L, BMI of ≥ 25, WHR > 0.95, and low physical activity (> 600 MET•min•wk^-1^) [5, 11]. The number of risk factors represents the sum number of the former condition that is present in each participant.

### Statistical analysis

The obtained data was analyzed using the SPSS (Statistical Package for the Social Sciences, version 17.0, SPSS Inc., Chicago, IL, USA), and presented as means ± SD for continuous variables and as number and frequencies for categorical variables. The frequency of CV disease risk factors per gender were compared using Pearson’s chi square. Associations of lipoprotein profile indices, obesity, glucose and WHR with CV disease risk factors were evaluated by Pearson’s correlation. For all statistical analyses, the significance level was set at P < 0.05.

## Results

A total of 348 students with a mean age 20.7 ± 1.7 years and a male:female ratio of 39:61, agreed to participate in the study. Table 1 shows the health-related fitness, hematological, and biochemical indices of the participants as total and divided by gender. Significant difference was observed between males and females in all body parameters (P < 0.001), glucose and Hematocrit (HCT) levels (P < 0.05).

**Table 1:** Body, Hematological and Biochemical Variables of the Participants According to Gender

Variable	Total (mean ± SD)	Gender		
		Male (mean ± SD)	Female (mean ± SD)	P-value
Body height	167.69 ± 9.16	174.97 ± 7.02	162.75 ± 6.86	0.000
Body weight	65.35 ± 14.83	74.45 ± 14.48	59.21 ± 11.56	0.000
Body fat%	24.93 ± 6.20	22.00 ± 6.47	26.78 ± 5.26	0.003
Body water%	54.78 ± 4.51	56.93 ± 4.72	53.43 ± 3.80	0.003
Body muscle%	40.10 ± 4.81	44.84 ± 3.59	37.11 ± 2.53	0.000
BMI	23.12 ± 4.10	24.32 ± 4.31	22.31 ± 3.76	0.000
WHR	0.79 ± 0.07	0.83 ± 0.06	0.76 ± 0.06	0.000
Bone mass	7.57 ± 2.13	9.91 ± 1.29	6.12 ± 0.86	0.948
RBC	5.00 ± 0.55	5.48 ± 0.38	4.69 ± 0.39	0.298
HGB	13.72 ± 1.56	15.29 ± 0.91	12.71 ± 0.93	0.083
HCT	43.27 ± 4.67	47.81 ± 3.09	40.37 ± 2.82	0.020
MCV	86.81 ± 6.36	87.34 ± 5.82	86.46 ± 6.67	0.341
Glucose	4.54 ± 0.49	4.68 ± 0.58	4.45 ± 0.40	0.000
Cholesterol	4.00 ± 0.74	3.97 ± 0.79	4.01 ± 0.71	0.140
TG	1.00 ± 0.52	1.15 ± 0.59	0.90 ± 0.44	0.427
HDL	1.15 ± 0.33	1.02 ± 0.29	1.22 ± 0.33	0.053
LDL	2.41 ± 0.62	2.42 ± 0.65	2.40 ± 0.60	0.156

Values are in mean ± SD. RBC: red blood cells; HGB: hemoglobin; HCT: hematocrit; MCV: mean cell volume; TG: triglyceride; HDL: high density lipoprotein; LDL: low density lipoprotein; BMI: body mass index; WHR: waist and hip ratio.

**Figure 1:**
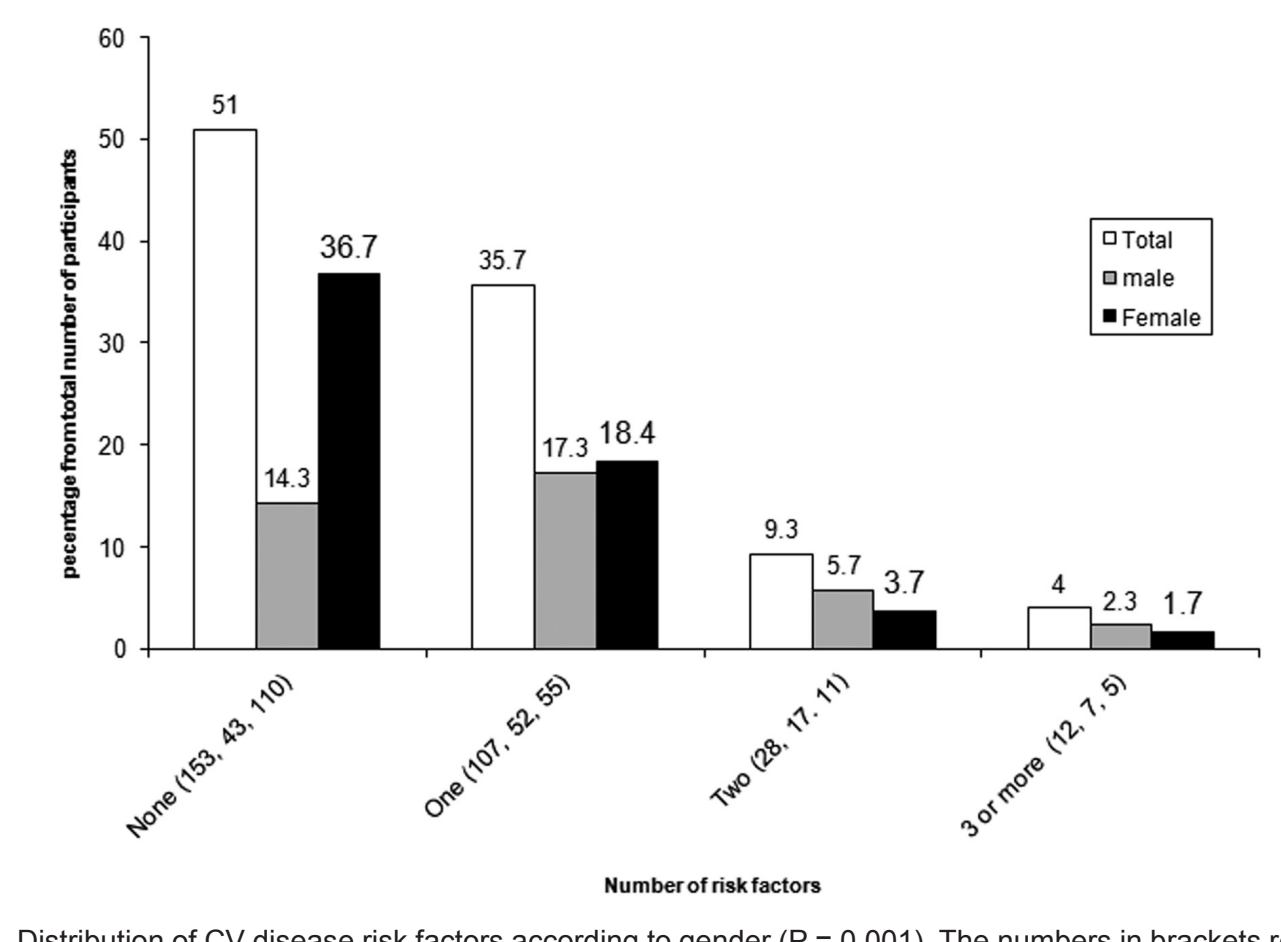
Distribution of CV disease risk factors according to gender (P = 0.001). The numbers in brackets represent the number of participants having the specified figure of CV disease risk factors, while the value above the each column represent the percentage of participants having the particular number of CV disease risk factors.

Table 2 shows the frequency of each CVD risk factors as total and divided by gender. The most common CV disease risk factors were low HDL-C (29.6%), and excess weight (BMI ≥ 25) (25.5%) with overweight and obesity rates of 18.9% and 6.6%, respectively, followed by high TG levels (8.6%).The prevalence of low HDL-C (P = 0.000), excess obesity (P = 0.001) and high TG (P = 0.002), were greater in the males than the females. The percentage of subjects with no and at least 1 CV disease risk factor were 51% and 49%, respectively, with 63.8% and 39.2% of the males and females, respectively, having at least 1 risk factor. In fact, males were more likely to have CV disease risk factors as compared to females (Fig. 1; P = 0.0001). The proportion of subjects having 1, 2, and ≥ 3 CV disease risk factors were 35.7, 9.3 and 4%, respectively.

**Table 2:** Assessment of CVD Risk Factors Among University Students as Total and Divided by Gender

Variable	Total n (%)	Gender		Pearson Chi-Square
		Male n (%)	Female n (%)	
Glucose				
≤ 5.89 mmol/L	313 (99.40)	120 (38.10)	193 (61.30)	0.074
> 5.89 mmol/L	2 (0.60)	2 (0.60)	0 (0.00)	
Cholesterol				
< 5.2 mmol/L	299 (94.90)	116 (36.8)	183 (58.10)	0.513
5.2 - 6.2 mmol/L	13 (4.1)	4 (1.3)	9 (2.9)	
> 6.2 mmol/L	3 (1.0)	2 (0.60)	1 (0.30)	
TG				
≤ 1.7 mmol/L	288 (91.40)	104 (33.00)	184 (58.40)	0.002
> 1.7 mmol/L	27 (8.60)	18 (5.70)	9 (2.90)	
HDL				
> 1 mmol/L	222 (70.50)	68 (21.60)	154 (48.90)	0.000
≤ 1 mmol/L	93 (29.50)	54 (17.10)	39 (12.40)	
LDL				
< 3.4 mmol/L	251 (93.00%)	94 (34.80%)	157 (58.10%)	0.895
3.4 - 4.1 mmol/L	17 (6.3%)	7 (2.60%)	10 (3.70%)	
> 4.1 mmol/L	2 (0.7%)	1 (0.4%)	1 (0.4%)	
BMI				
< 18.5	30 (9.40)	6 (1.90)	24 (7.50)	0.001
18.5 - 24.99	207 (65.10)	76 (23.90)	131 (41.20)	
25 - 29.99	60 (18.90)	33 (10.40)	27 (8.50)	
≥ 30	21 (6.60)	13 (4.10)	8 (2.50)	
WHR				
< 0.95 (M), ≥ 0.9 (F)	319 (99.10)	128 (39.80)	191 (59.30)	0.351
> 0.95 (M), ≥ 0.9 (F)	3 (0.90)	2 (0.60)	1 (0.30)	
Total PA				0.255
Low	45 (13.2)	14 (10.7)	31 (14.8)	
Moderate	98 (28.8)	34 (26.0)	64 (30.6)	
High	197 (57.9)	83 (63.4)	114 (54.5)	

As in Table 3, percent body fat, percent bone, glucose, total cholesterol, TG, LDL-C, BMI, and WHR, correlated positively with the number of CV disease risk factors in the males, females, and the combined genders (range of R^2^: 0.17 to 0.603). On the other hand, negative correlations were detected between physical activity, body water and percent body muscle, and HDL-C, with the number of CV disease risk factors in the males, females, and the combined genders (range of R^2^: -0.239 to -0.412). Finally, red blood cells (RBCs), hemoglobin (HGB), HCT, and mean cells volume (MCV) were positively correlated with the number of CV disease risk factors in the study participants as a total, but not per individual gender (Table 3).

**Table 3:** Pearson’s Correlation Between Number of CVD Risk Factors Present and Other Parameters

Number of risk factors versus	Pearson's correlation coefficient		
	Total	Males	Females
Body Fat%	0.216**	0.372**	0.294**
Body water%	-0.217**	-0.372**	-0.299**
Body muscle%	-0.001	-0.365**	-0.244**
Bone mass	0.358**	0.433**	0.176*
BMI	0.389**	0.423**	0.289**
WHR	0.263**	0.335**	0.061
RBC	0.190**	0.089	-0.007
HGB	0.257**	0.106	0.121
HCT	0.214**	0.097	0.007
MCV	0.014	-0.016	0.012
Glucose	0.123*	0.197*	-0.063
Cholesterol	0.173**	0.271**	0.113
TG	0.509**	0.573**	0.385**
HDL-C	-0.496**	-0.564**	-0.394**
LDL-C	0.236**	0.230*	0.248**
Total PA	-0.336**	-0.228*	-0.482**

** Correlation is significant at the 0.01 level; * Correlation is significant at the 0.05 level (2-tailed).

## Discussion

Results of this study show high prevalence of several modifiable CV disease risk factors among college students with 49% had at least 1 risk factor. The presence of risk factors was associated with increases in percent body fat and bone, glucose, total cholesterol, TG, LDL-C, BMI, and WHR. On the other hand, percentages of physical activity, body water and muscle, HDL-C associated inversely with CV disease risk factors.

In the current study, the most frequent risk factors among the students in chronological order were reduced HDL-C (29.5%), excess obesity (18.9%), physical inactivity (13.2%), and elevated TG (5.1%). The occurrence of undesirable blood lipid profile was also reported in Columbian (low HDL-C = 13.3%) [16], Chilean (high TC = 29.2% and LDL-C = 16.2%, and low HDL-C = 5%) [28], and Brazilian (high TC = 17.7%, LDL-C = 10.2%, and TG = 11.1, and low HDL-C = 11.1%) [18] college students. However, excess obesity (as defined by abdominal circumference or BMI) was prevalent risk factor in the majority of the found studies. It was 45.5 and 24.3% of the Chilean men and women, respectively [19], 40 and 23% of the Greek men and women, respectively [29], 18% in the Brazilians [18], 15.4% in the Portuguese [15], 11.2% in the Columbians %) [16], and 7.27% in the Srbijein males [20]. The most common risk factors found in Hungarian university students were increased waist circumference, and TG and low HDL-C [30], whereas low HDL-C, and high TG were the most common among Latin American university students [16]. However, in our study, the prevalence of low HDL-C (29.5%) and high BMI (18.9%) were much greater, while the prevalence of high TG was comparable to previously reported [16, 20, 30-31]. The prevalence of physical inactivity observed in our study is within the range reported in other university student's populations [16, 20, 30-31].

The greater prevalence of risk factors (i.e low HDL-C, and elevated BM and TG) among the males as compared to females, in our study, was also found in studies from other countries. Differences between genders in the prevalence of risk factors were found also in Portuguese students as the occurrence of hypercholesterolemia was in 15.6 vs. 2.1% and of hypertension was found in 13.7 vs. 3.5% of the males and females, respectively [15]. The prevalence of high BMI and abdominal obesity were greater in the university males than the females from Srbija whereas smoking cigarettes was similar in both genders and physical inactivates was greater in the females than the males [20]. The occurrence of abdominal obesity (m: 40 vs. f: 23%) and overweight (m: 33.4 vs. f: 27.1%) were was also greater in Greek males and were best indicators for hypertension and dyslipidaemia, respectively, in both genders [29]. Similar to our study, the male students from Chile were more obese than the females [19]. Abdominal obesity, overweight, and undesirable blood lipid values occurred in greater rates in the males than females in Hungarian students [30]. Additionally, BMI values correlated with waist circumference, total cholesterol, LDL-C, apolipoprotein B, and systolic and diastolic blood pressures in both groups and with HDL-cholesterol in the females [30]. On the other hand, our study showed that the prevalence of physical inactivity was higher in female students than male students. Similar to our finding, high frequency of physical inactivity was reported in female Portuguese [15] and Chilean [19] students than male students. Discrepancies among studies of CVD risk factors are related to several factors including variations in age, geographic distribution, ethnic backgrounds, environment, lifestyles, and genetics of the studied populations. In addition, the variations in the prevalence of risk factors in the different populations and genders highlight the importance of studying these factors in other populations and indicate that each country may need different intervention programs for its population and for each gender.

Percent body fat correlated positively, whereas Percent body muscles correlated negatively with the number of CVD risk factors, indicating the importance of body composition to CV health. This is in accordance with the fact that increased body fat, along with reduced muscles, indicates lower fitness status, which is associated with higher prevalence of CVD. Strong correlation between physical activity and CVD risk factors was reported in this study. This confirms the role of physical activity in prevention of CV diseases and in controlling blood lipid abnormalities, diabetes, obesity and hypertension [32].

Among the CV disease risk factors that were not investigated in this study are genetics factors. Several genes were found to modulate susceptibility to CVD including more than 100 loci [33, 34]. The prevalence of such risk factors among studied population will be a matter of our future research.

### Conclusions

Results of the current study indicate the high prevalence of several modifiable CV disease risk factors among young adult population. As recommended by several health-related organizations such as the World health organization (WHO) and the American College of Sports Medicine, ACSM), prevention and intervention programs of these risk factors should start as early in age as possible.
